# Supplementary material for: Development of an In Vitro Blood Vessel Model Using Autologous Endothelial Cells Generated from Footprint-Free hiPSCs to Analyze Interactions of the Endothelium with Blood Cell Components and Vascular Implants
Source: Cells. 2023 Apr 22;12(9):1217. doi: 10.3390/cells12091217 (PMC10177426; doi:10.3390/cells12091217)
Supplement: Supplementary file 1 [file cells-12-01217-s001.zip › cells-2263282-supplementary.pdf]

cells

MDPI

Supplementary Material

Development of an In Vitro Blood Vessel Model Using Autologous Endothelial Cells Generated from Footprint-Free hiPSCs to Analyze Interactions of the Endothelium with Blood Cell Components and Vascular Implants

Josefin Weber, Marbod Weber, Adrian Feile, Christian Schlensak and Meltem Avci-Adali *

Department of Thoracic and Cardiovascular Surgery University Hospital Tuebingen, , Calwerstraße 7/1, 72076 Tuebingen, Germany
* Correspondence: meltem.avci-adali@uni-tuebingen.de

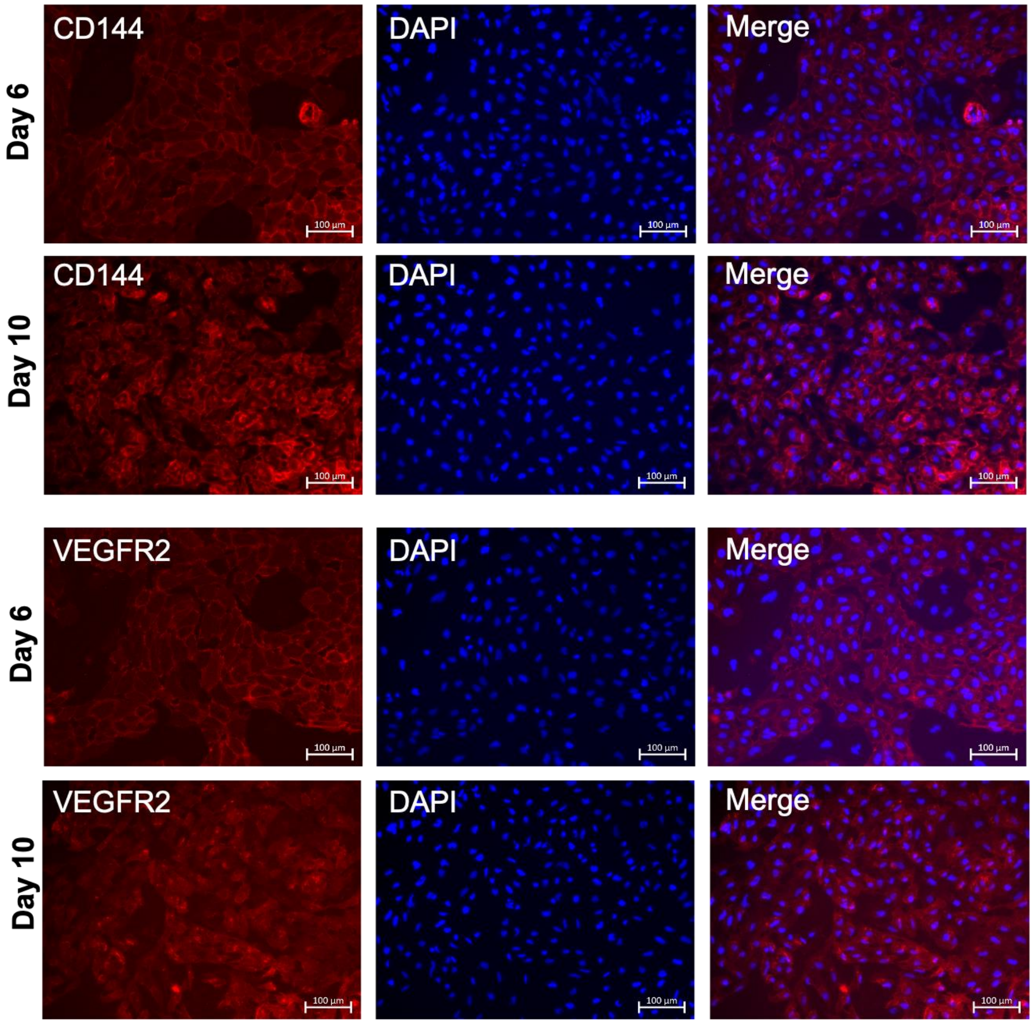

Figure S1. Representative immunofluorescence microscopy images of hiPSC-ECs. The cells were stained with PE-labeled CD144- and VEGFR2-specific antibodies on day 6 (before separation) and 10 of differentiation. Scale bars represent 100 µm.

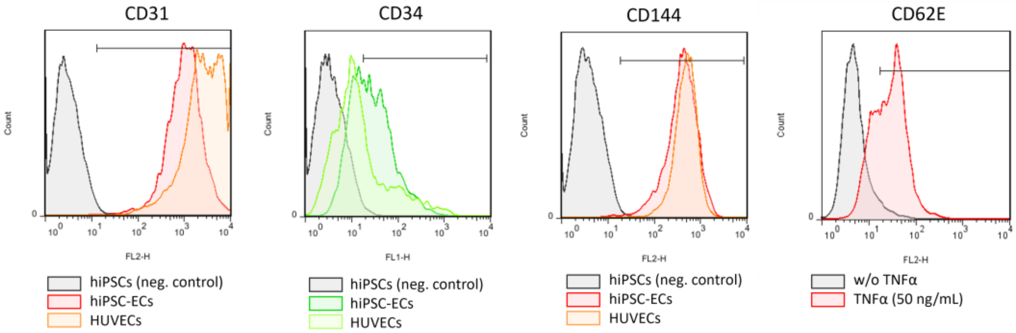

Figure S2. Characterization of hiPSC-ECs. Representative flow cytometry histograms of the markers used for cell characterization (CD31, CD34, CD144, and CD62E).

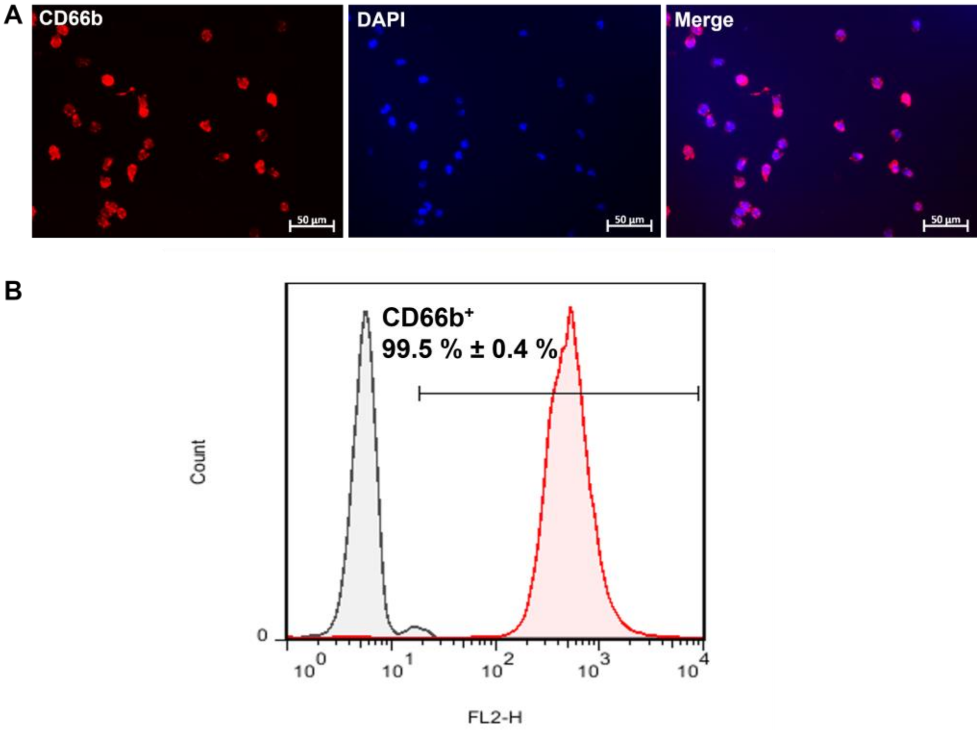

Figure S3. Characterization of isolated granulocytes from human blood. (**A**) Representative immunofluorescence microscopy images of granulocytes stained with a CD66b-specific antibody. Scale bars represent 50 µm. (**B**) Flow cytometry analysis of isolated neutrophil granulocytes expressing CD66b ($n = 3$).

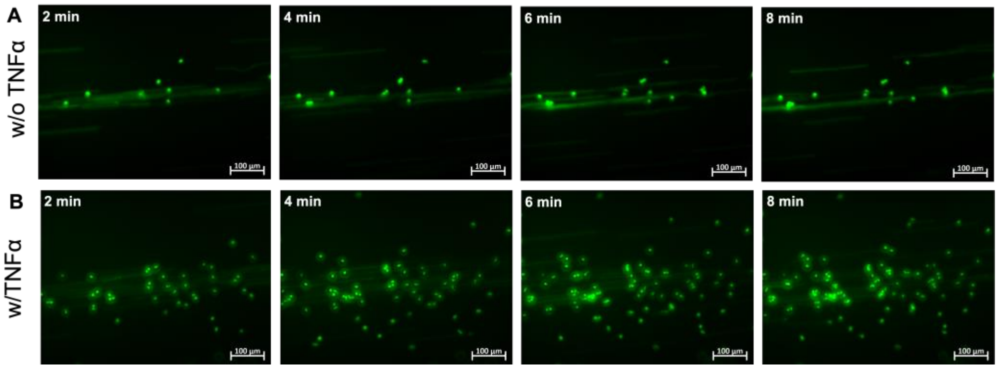

Figure S4. Interaction of fluorescently labeled granulocytes with hiPSC-ECs under flow conditions. (**A**) Interaction of fluorescently labeled granulocytes (calcein AM, green) with unstimulated and (**B**) 50 ng/ml TNF-α-stimulated hiPSC-ECs in a PDMS model during perfusion. Granulocytes were perfused with a flow rate of 150 µL/min for 10 min over the ECs, corresponding to a shear stress of 0.4 dyne/cm². Images were acquired after 2, 4, 6, and 8 min of perfusion.
